# RAG2 localization and dynamics in the pre-B cell nucleus

**DOI:** 10.1371/journal.pone.0216137

**Published:** 2019-05-10

**Authors:** William Rodgers, Jennifer N. Byrum, Destiny A. Simpson, Walker Hoolehan, Karla K. Rodgers

**Affiliations:** 1 Department of Biochemistry and Molecular Biology, University of Oklahoma Health Sciences Center, Oklahoma City, Oklahoma, United States of America; 2 Department of Microbiology and Immunology, University of Oklahoma Health Sciences Center, Oklahoma, United States of America; Universität Stuttgart, GERMANY

## Abstract

RAG2 of the V(D)J recombinase is essential for lymphocyte development. Within the RAG2 noncore region is a plant homeodomain (PHD) that interacts with the modified histone H3K4me3, and this interaction is important for relieving inhibition of the RAG recombinase for V(D)J recombination. However, the effect of the noncore region on RAG2 localization and dynamics in cell nuclei is poorly understood. Here, we used cell imaging to measure the effect of mutating the RAG2 noncore region on properties of the full length protein. We measured GFP-labeled full length RAG2 (FL), the RAG2 core region alone (Core), and a T490A mutant in the noncore region, which has unique regulatory properties. This showed that FL, T490A, and Core localized to nuclear domains that were adjacent to DAPI-rich heterochromatin, and that contained the active chromatin marker H3K4me3. Within the RAG2-enriched regions, T490A exhibited greater colocalization with H3K4me3 than either FL or Core. Furthermore, colocalization of H3K4me3 with FL and T490A, but not Core, increased in conditions that increased H3K4me3 levels. Superresolution imaging showed H3K4me3 was distributed as puncta that RAG2 abutted, and mobility measurements showed that T490A had a significantly lower rate of diffusion within the nucleus than either FL or Core proteins. Finally, mutating Trp^453^ of the T490A mutant (W453A,T490A), which blocks PHD-dependent interactions with H3K4me3, abolished the T490A-mediated increased colocalization with H3K4me3 and slower mobility compared to FL. Altogether, these data show that Thr^490^ in the noncore region modulates RAG2 localization and dynamics in the pre-B cell nucleus, such as by affecting RAG2 interactions with H3K4me3.

## Introduction

V(D)J recombination generates antigen receptor (AR) genes in developing lymphocytes by a cut-and-paste recombination reaction from component genes in AR loci [1, 2]. The large array of gene combinations that follow from the V(D)J recombination is the basis for sequence diversity of the antigen binding receptors in the immune system. The V(D)J recombinase, which consists of the RAG1 and RAG2 proteins, function by creating DNA double strand breaks at select recombination signal sequences (RSS) that are located at the border of each gene segment in the AR loci. A critical step in this process is the selective binding of the RAG proteins to an RSS over the vast array of accessible DNA in the genome. Importantly, the genome also contains numerous RSS-like sites, where RAG binding and DNA cleavage activity can result in oncogenic chromosomal translocations [3–5]. Thus, properties that affect targeting of the RAG complex to the genome have important consequences regarding genomic integrity.

Transcription facilitates V(D)J recombination, since conditions that increase transcription also increase V(D)J recombination [6]. Transcriptionally active regions of the genome occur in euchromatin that is biochemically marked by distinct modifications of core histones, such as trimethylation of histone H3 on lysine 4 (H3K4me3) [7]. This contrasts with the transcriptionally silent heterochromatin, which is highly condensed and marked by repressive histone markers, such as trimethylation of histone H3 at lysine 9 (H3K9me3). Within the nucleus, heterochromatin resides principally at the periphery, and inactive AR alleles that do not undergo recombination are also positioned at the nuclear periphery [8, 9]. Nevertheless, topological studies have shown that condensed chromatin containing inactive histone markers also occurs throughout the nuclear interior, abutted with transcriptionally active chromatin labeled with H3K4me3 and RNA polymerase II [10].

Placement of the active chromatin marker H3K4me3 near AR loci often facilitates V(D)J recombination, since binding of RAG2 to H3K4me3 relieves an autoinhibition of the RAG complex to activate endonucleolytic activity for V(D)J recombination [11–14]. While interactions between RAG2 and H3K4me3 are thought to assist in recruiting the RAG complex to active AR loci, ChIP-seq studies showed it also recruits the complex to open chromatin sites throughout the genome [15, 16].

RAG2 interacts with H3K4me3 through a plant homeodomain (PHD) in the RAG2 noncore region [17–19]. The PHD is a zinc-binding module that functions as an epigenetic reader, typically recognizing unmodified or methylated histone H3 [20]. In RAG2, the PHD is present in the noncore region and encompasses residues 414–487 of the 527-residue full-length protein [18, 19, 21]. Overall, the noncore region of RAG2 appears to play multiple roles in regulating RAG function [1, 2]. For example, RAG2 degradation is activated at the G1/S border of the cell cycle through CDK2/Cyclin A phosphorylation of Thr^490^, which is adjacent to the PHD. The Thr^490^-dependent regulation of RAG2 degradation restricts RAG2 expression and V(D)J recombination to the G0/G1 phases of the cell cycle [22]. Besides affecting RAG2 degradation, Thr^490^ may have other impacts on RAG2 structure and function, since the T490A mutation in contrast to wild type RAG2 does not undergo nuclear export in pre-B cells following genotoxic stress [23].

Localization studies of RAG2 expressed in pre-lymphoid cells or transiently-expressed in non-lymphoid cells demonstrated that RAG2 localizes in the nucleoplasm and is secluded from nucleoli [9, 23–26]. Importantly, these studies did not show the properties of RAG2 localization relative to discrete epigenetic markers, nor the dynamics of RAG2 within the nucleus. Altogether, properties of RAG2 noncore that regulate its targeting to H3K4me3, the distribution of these sites within the genome measured in intact cells, and the effect of these interactions on RAG2 dynamics within the nucleus, remain poorly understood.

To resolve the role of the RAG2 noncore region in determining RAG2 localization and dynamics, we measured GFP-labeled wild type and noncore mutant RAG2 proteins in pre-B cell nuclei using a combination of separate fluorescence microscopy methods. Our data show that the RAG2 proteins localized to intranuclear domains that abutted DAPI-enriched heterochromatin regions throughout the nucleus. Within the RAG2-enriched domains, a T490A mutant of the RAG2 noncore domain exhibited the greatest colocalization with H3K4me3, and this coincided with a slower intranuclear mobility compared to labeled wild-type and Core-only truncation mutant of RAG2. Importantly, mutating Trp^453^, which is important for RAG2 PHD interactions with H3K4me3, in the T490A mutant to produce the double mutant W453A,T490A resulted in a decrease in RAG2 colocalization with H3K4me3, and an increase in mobility to values similar to that of the wild-type RAG2. Altogether, our findings show that RAG2 is sequestered from heterochromatin to nuclear channels that contain active chromatin, and Thr^490^ modulates RAG2 interactions with intranuclear binding partners within the channels, such as PHD-dependent interactions with H3K4me3, to affect RAG2 localization and dynamics.

## Materials and methods

### Cell culture and reagents

v-abl *RAG2*^*-/-*^ pro-B cells (63–12), a v-abl-transformed mouse *RAG2*^*-/-*^ pro-B cell line [27], were maintained in complete media containing RPMI with 10% FCS, 0.1% 2-mercaptoethanol, 2% sodium pyruvate, 1% nonessential amino acids and 10% fetal bovine serum. cDNA encoding eGFP-labeled RAG2 constructs have been previously described [23]. These consisted of wild-type full length (FL) RAG2, Core (consisting of residues 1 through 377), and two full length RAG2 mutants, T490A and W453A,T490A. Each RAG2 was fused to eGFP at the N-terminus (GFP-RAG2). Stable clones expressing GFP-RAG2 fusion proteins were generated by limiting dilution of transfected cells in complete media containing G418 at a final concentration of 1.5 mg/ml, followed by flow cytometry to enrich GFP^+^ cells. Following selection, the cells were maintained in complete media containing 0.5 mg/ml G418. Immunoblot of cell lysates showed that GFP-RAG2 was not overexpressed relative to endogenous RAG2 in v-abl pre-B cells treated with STI-571 (S1A Fig), and that each GFP-RAG2 construct exhibited comparable V(D)J recombinase activity in an extrachromosomal plasmid recombination assay (S1B Fig).

For treatment of cells with 2,4-pyridinedicarboxylic acid (PDA, Sigma-Aldrich, St. Louis, MO), 10^6^ cells were cultured overnight in 1.0 mL of media containing 10.0 mM PDA that was added from a 200 mM stock in 1M Tris-HCl pH 9.0. To induce RAG1 expression, cells were cultured overnight at a density of 10^6^ cells/ml in 1.0 ml of media containing 5 μM STI-571 (Cayman Chemical, Ann Arbor, MI) that was added from a stock solution containing drug at a concentration of 10 mg/ml in DMSO. Antibodies for labeling and detection of proteins were mouse monoclonal antibody to H3K4me3 (clone MABI 0304, Active Motif, Carlsbad, CA), rabbit polyclonal antibody to H3K9me3 (A-4036, Epigentek, Farmingdale, NY), and rabbit monoclonal antibody to cyclin D1 (clone EPR2241, Abcam, Cambridge, MA). Secondary antibodies were Cy3-conjugated anti-rabbit and Alexa647-conjugated anti-mouse IgG, each from goat (Jackson Immunoresearch, West Grove, PA). Monoclonal antibody to RAG2 (clone EPRAGR239, Abcam, Cambridge, MA) was used for detection in immunoblotting experiments.

### Pull-down assay

HEK-293T cells were transfected with plasmids encoding GFP-RAG2 using Fugene 6 (Promega, Madison, WI). Cells were harvested 24 hours post-transfection by washing twice with chilled PBS (4°C), followed by resuspending in buffer containing 20 mM Tris (pH 7.6), 200 mM NaCl, 2 mM MgCl_2_, 0.5 mM DTT, 0.5% glycerol, 1mM PMSF, and 2 μg/mL aprotinin. Next, the cells were lysed by sonication, followed by centrifugation (6800 x *g*, 3 min at 4°C). Cleared cell lysate was added to streptavidin-coupled magnetic beads (Thermo Fisher Scientific, Waltham, MA) coated with biotinylated peptide that corresponded to the N-terminal 20 amino acids of either histone H3, H3K4me3, or H3K9me3 (EpiCypher, Research Triangle Park, NC). Prior to addition of the cell lysate, the streptavidin-coupled beads were incubated overnight at 4°C with 12 μM biotinylated peptide, followed by washing with buffer containing 10 mM Tris-HCl, 150 mM NaCl, 0.005% Tween 20, pH 7.5. The peptide coated beads were subsequently incubated with lysate overnight at 4°C, then washed and bound proteins eluted in SDS sample buffer, separated by SDS-PAGE, and measured by immunoblotting for RAG2.

### Cell staining and fluorescence imaging

Samples were prepared by first seeding 10^6^ cells onto a 15 mm #1.5 round glass coverslip coated with poly-L-lysine (Sigma-Aldrich). Following seeding, the samples were fixed by incubating in PBS containing 4% paraformaldehyde for 30 min at room temperature, then permeabilized using 0.1% TX-100 in PBS with 10 mM glycine (PBS-glycine). For immunostaining, fixed and permeabilized cells were incubated with 0.5–1.0 μg of primary antibody in 500 μl of PBS-glycine at room temperature for 45 min, followed by washing and incubation with 2.0 μg of secondary antibody for 45 min at room temperature. Following immunostaining, the samples were labeled with DAPI using a 300 nM solution in PBS-glycine for 5 min at room temperature. The samples were mounted using ProLong Diamond Antifade (Thermo Fisher, Waltham, MA).

Confocal microscopy was performed using a Zeiss LSM-710 equipped with a 63x 1.4 NA objective (OMRF Imaging Core). Structured Illumination Microscopy (SIM) was performed using DeltaVision OMX-SR system (OMRF Imaging Core).

### Cell Image Analysis

Image processing and quantification were performed using iVision (BioVision Technologies, Exton, PA). Protein colocalization was quantified using Pearson’s correlation coefficient (ρ), calculated as described [28]. For determining ρ, a DAPI image of the sample was used to create a mask that restricted the region of interest (ROI) to the nucleus. Following subtraction of the background, the ROI in each channel was summed for determination of the Pearson correlation coefficient. Colocalization was measured individually for each cell in the field.

### Fluorescence recovery after photobleaching (FRAP)

FRAP was performed using a 40x water 1.2 NA objective on a Zeiss LSM-710 equipped with a thermocontrolled chamber to maintain the samples at 37°C with 5% CO_2_. Cells were maintained in RPMI (without phenol red) containing 0.5% FCS in 35 mm dishes with a sealed coverslip on the bottom (MatTek, Ashland, MA). Cells were adhered onto the coverslip prior to imaging using poly-L-lysine. For FRAP, a 80 x 80 ROI (zoom = 2) was selected within a field of cells. A 10 x 10 circle within the nucleus was selected for photobleaching. Bleaching was performed using the 488 nm line on an argon laser at 100% power. Images were acquired every 0.1 s, typically for 60 frames. The recovery within the bleached region at each time point was normalized using the equation:

$$R=A_{o}I_{t}/A_{t}I_{o}$$ (1)

where A_*o*_ is the total intensity of the nucleus in the prebleach image, A_*t*_ is the total intensity of the nucleus at time point *t*, I_*o*_ is the intensity of the bleached region in the prebleach image, and I_*t*_ is the intensity of the bleached region at time point *t*. R therefore represents the ratio F_*t*_:F_*o*_ corrected for the decay of fluorescence from image acquisition during the experiment, where F_*t*_ is the fluorescence intensity of the photobleached spot at time *t*, and F_*o*_ is the fluorescence intensity of this region in the prebleach image. Determined values for R were imported into Prism and plotted versus time following photobleaching. Rate constant and percent recovery values were determined from a one-component exponential fit of recovery data. Each trial consisted of 8 to 12 FRAP measurements. The averaged data from three or more trials was used for curve fitting to determine the rate and amount of recovery of each clone, and the standard error of the mean (SEM) for each time point.

### Statistical analysis

Analysis was performed using Prism 5 software (GraphPad Software, La Jolla, CA). SEM was calculated using the average of each of three or more independent trials. Significance was tested by null hypothesis using a two-tailed Student’s *t*-test, where ****, *p* < 0.0001, ***, *p* < 0.001, **, *p* < 0.01, and *, *p* < 0.05, ns (not significant) and *p* ≥ 0.05.

## Results

### RAG2 localizes to DAPI-poor regions that contain active chromatin

**Fig 1:**
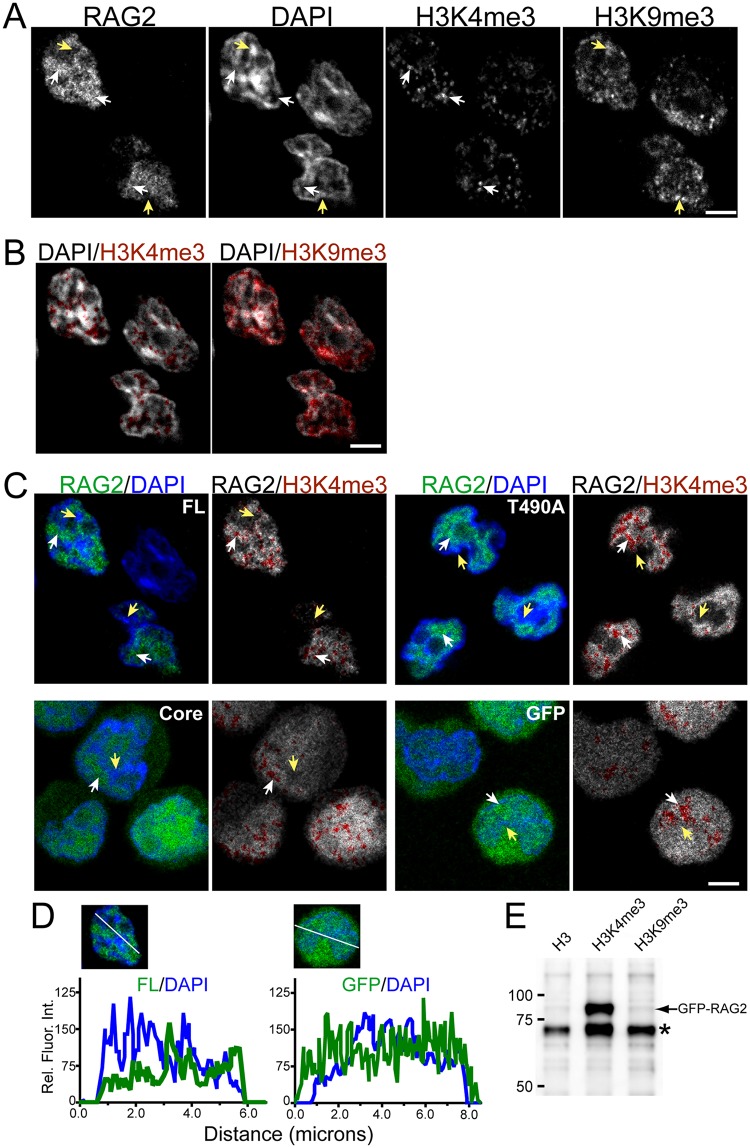
Visualization of RAG2 localization to DAPI-poor regions of the pre-B cell nucleus. **(A)** Confocal images of DAPI-stained nuclei of pre-B cells expressing GFP-RAG2, and immunostained with antibodies to H3K4me3 and H3K9me3. The white arrows indicate corresponding regions of RAG2 and H3K4me3 labeling; the yellow arrows indicate regions of DAPI enrichment that also contained H3K9me3. **(B)** Overlay of labeled H3K4me3 *(left)* and H3K9me3 *(right)* on DAPI. The labeled histones are red, and the DAPI is grayscale. **(C)** Merge *(left)* and overlay images *(right)* of cells expressing FL, T490A, Core, or GFP control not fused to RAG2. The merge images were generated using the DAPI *(blue)* and GFP *(green)* channels of each image. The overlays are labeled H3K4me3 *(red)* on the GFP channel *(grayscale)*. The white and yellow arrows indicate GFP- and DAPI-enriched domains, respectively. Note that FL in (C) is the same field shown in (A). The white bar in (A), (B), and (C) represents 3 μm. **(D)** Plots of the fluorescence intensities in the GFP (green) and DAPI (blue) channels, measured along the white line in the respective images of a cell that expressed either GFP-FL *(left)* or GFP alone *(right)*. **(E)** Pull-down assay of GFP-FL by modified and unmodified H3 N-terminal peptides. RAG2 was detected by immunoblotting with rabbit monoclonal antibody to RAG2. Molecular weights, in thousands, are indicated on the left. The asterisk indicates a nonspecific band.

In Fig 1A are representative confocal images of *Rag2*^*-/-*^ 63–12 cells expressing GFP-RAG2 FL, and co-stained with DAPI and antibodies to H3K4me3 and H3K9me3. The white arrows indicate examples of RAG2-labeled domains within the nucleus, which also contained H3K4me3. In contrast, DAPI appeared concentrated in regions adjacent to and non-overlapping with the RAG2 domains (yellow arrows), and these regions contained significantly more H3K9me3 than H3K4me3 (Fig 1B).

In addition to the FL RAG2, we also expressed in 63–12 cells a GFP-labeled T490A mutant of RAG2, and GFP-labeled Core RAG2. Finally, GFP not fused to RAG2 served as control for localization properties specific to the RAG2 protein. Our rationale for studying the T490A mutant was that this protein, in comparison to FL, exhibits unique nuclear export properties in cells following genotoxic stress [23], and this may reflect increased interactions between T490A and other nuclear proteins, such as modified histones.

In Fig 1C are pre-B cells that expressed GFP-labeled FL, T490A, Core, or GFP alone. The data are represented as a merge of the GFP and DAPI images *(left)*, and as overlay *(right)* of H3K4me3 staining (red) on the GFP (grayscale). In contrast to the RAG2 fusion proteins, the unfused eGFP showed a diffuse labeling between both the nucleus and cytoplasm. eGFP not concentrating in the nucleus is expected since it lacks a discrete nuclear import signal, yet is small enough to passively diffuse into the nucleus from the cytoplasm through the nuclear pore. As we previously reported, the FL protein exhibited slightly more cytoplasmic localization relative to T490A even in the absence of DNA damaging conditions [23]. Nevertheless, both proteins are predominantly nuclear. In this study, the localization properties of only the nuclear pool of RAG2 was analyzed (see Methods). Within the nucleus, both the T490A and Core were similar to FL by being localized to nuclear domains containing H3K4me3 (Fig 1C, white arrows) that were adjacent to and depleted from DAPI-enriched domains (yellow arrows). However, when not fused to RAG2, the GFP was relatively homogeneous, with exclusion from the heterochromatin-enriched regions appearing less acute than observed for the FL, T490A, and Core proteins. For example, in Fig 1D are plots of the fluorescence intensity of GFP and DAPI along a line drawn across cells that expressed either GFP-FL or GFP not fused to RAG2. These data show that FL exhibited relatively weak labeling in areas with the greatest DAPI staining, but labeling by unfused GFP persisted in the DAPI-rich regions. Exclusion of the labeled RAG2 proteins from the heterochromatin domains relative to GFP alone may be due to either their larger size [10], or by the noncore region actively excluding RAG2 from heterochromatin. Finally, we observed pull-down of RAG2 from cell lysates by a 21-residue peptide corresponding to the H3 N-terminal tail containing the K4me3 modification (Fig 1E), indicative of interactions between RAG2 and H3K4me3 that can occur in the RAG2-labeled domains.

### The noncore region of RAG2 is necessary for a PDA-dependent increase in RAG2 colocalization with H3K4me3

**Fig 2:**
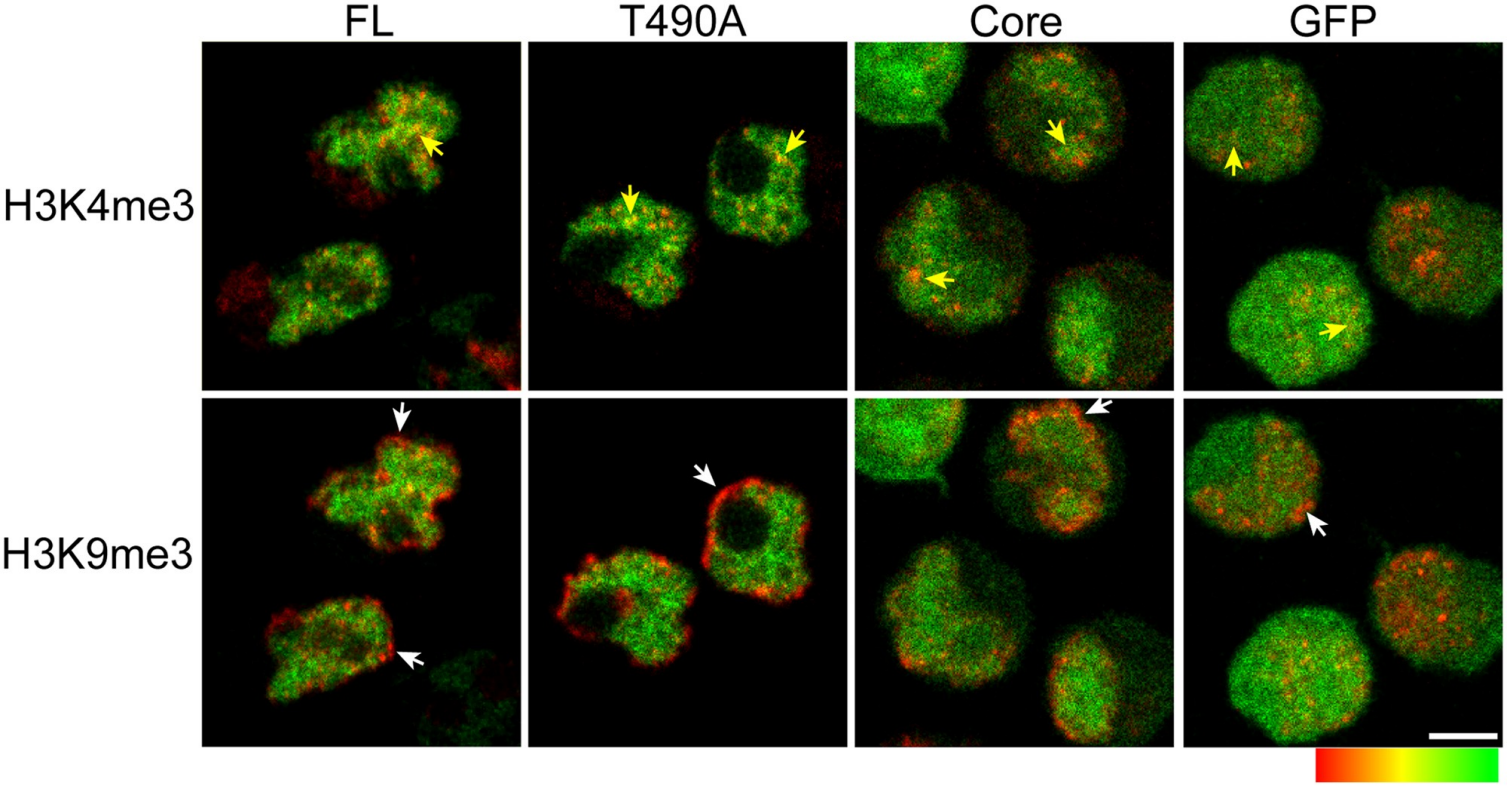
Visualization of RAG2 colocalization with H3K4me3 versus H3K9me3. Merge images of GFP-labeled FL, T490A, Core, or control GFP, with H3K4me3 *(top)* and H3K9me3 *(bottom)*. The spectrum shows the pseudocolor assignment, with green and red being GFP and labeled histone alone, respectively, and intermediate shades where the labels are correlated in regions of protein colocalization. Examples of correlated labeling are indicated by yellow arrows. The white arrows indicate heterochromatin stained with H3K9me3 and lacking labeled RAG2 in the nuclear periphery. The white bar indicates 3.0 μm.

Although removing the noncore region of RAG2 did not restrict RAG2 from DAPI-poor zones of the nucleus (Fig 1C), sequences within the noncore region may still affect RAG2 association with H3K4me3 sites within the DAPI-poor zones. For example, in merge images of the RAG2 constructs with H3K4me3 (Fig 2), the nuclear interior exhibits a range of correlation values, from red, which is mostly H3K4me3, to green, which is mostly the GFP label. This contrasts with merge images of the RAG2 constructs with H3K9me3 in Fig 2, which show segregation of the H3K9me3 to the nuclear periphery and the GFP label in the interior. Quantitation of RAG2 correlation with H3K4me3, measured in populations of double-labeled cells, may therefore show that removing either Thr490 or the noncore region affects RAG2 localization with the labeled H3K4me3 in the RAG2-enriched domains.

We quantitated the correlation of RAG2 with H3K4me3 by determining the Pearson’s correlation coefficient (ρ) for populations of labeled pre-B cells. Values for ρ will range from -1.0 for anti-correlated signals, to 0.0 for not correlated, and +1.0 for completely correlated. However, the maximum value for correlated signals detected in our system was approximately 0.70, which was determined by measuring ρ in cells double-labeled with separate secondary antibodies to detect H3K4me3 staining by a single primary antibody. This deviation from 1.0 likely reflects inherent properties of the system related to the labeling and its detection through separate channels in the imaging system.

**Fig 3:**
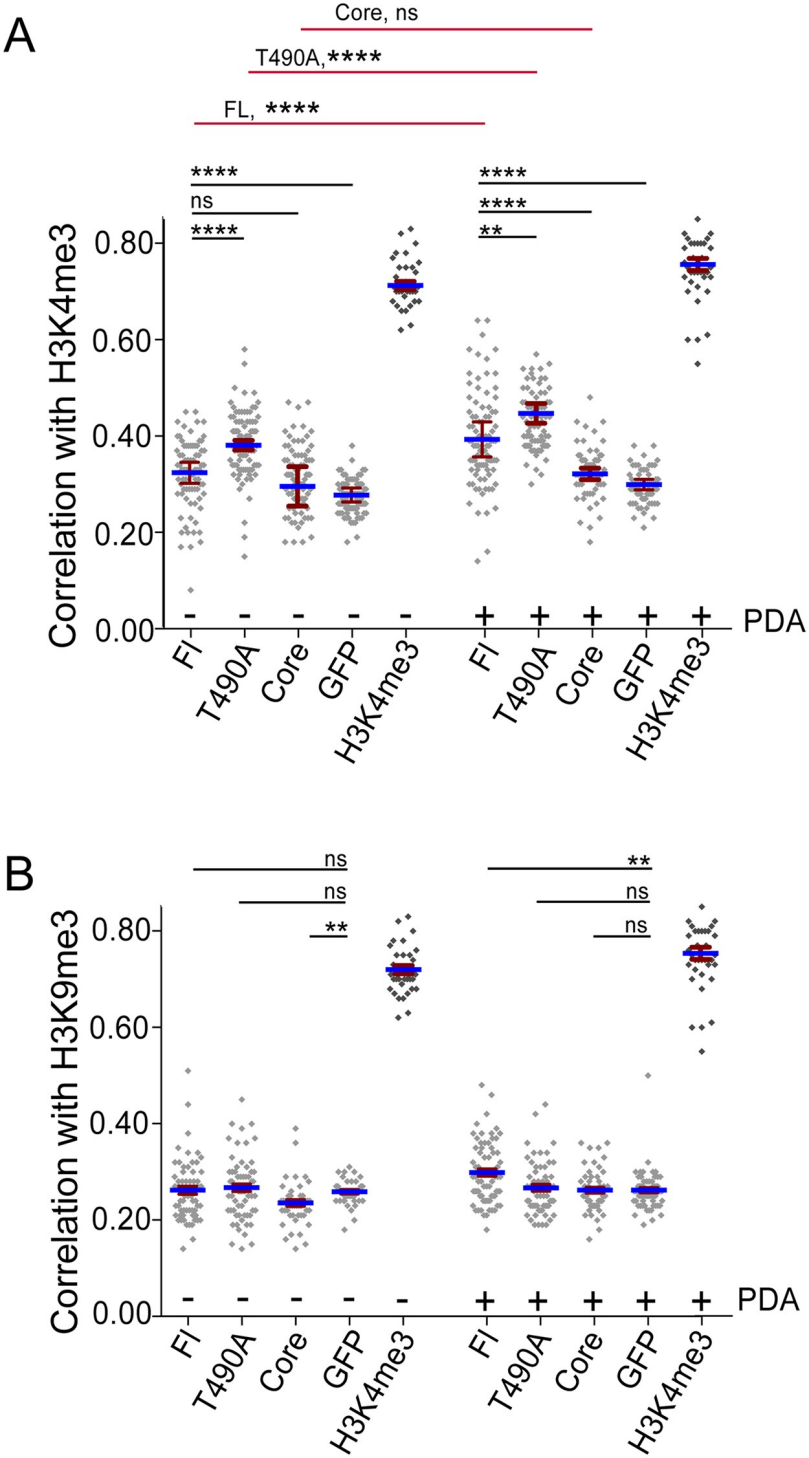
The T490A mutation increases RAG2 colocalization with H3K4me3. Pearson correlation values (ρ) for GFP-labeled RAG2 proteins and either H3K4me3 **(A)** or H3K9me3 **(B)**. Pre-B cells expressing the indicated GFP-RAG2 were co-stained with separate antibodies to H3K4me3 and H3K9me3. Secondary antibodies labeled with either Alexa 647 or Cy3 were used to detect the H3K4me3 and H3K9me3 labeling, respectively. The columns labeled H3K4me3 are a positive control consisting of cells labeled with rabbit antibody to H3K4me3 and co-stained with Alexa488- and Cy3-labeled antibodies to the primary antibody. Each symbol represents measurement of an individual nucleus. Indicated are the mean (blue lines) and SEM (error bars), calculated from the mean of three or more trials, each trial consisting of measurement of at least 30 cells. The red lines indicate significance tests between corresponding double-labeled samples, comparing cells with PDA treatment to control cells without PDA.

Plotted in Fig 3A are the results from the correlation analysis of the labeled RAG2 proteins and GFP with H3K4me3, with each point representing ρ measured for an individual cell. Summarizing, these data show that ρ averaged significantly larger for the T490A samples compared to FL and Core samples, suggesting this mutant increases or stabilizes interactions between RAG2 and H3K4me3. Interestingly, ρ values for FL and H3K4me3 averaged most similar to that of Core and H3K4me3, indicating that the wild type noncore region does not contribute significantly to steady-state colocalization of RAG2 with H3K4me3.

**Fig 4:**
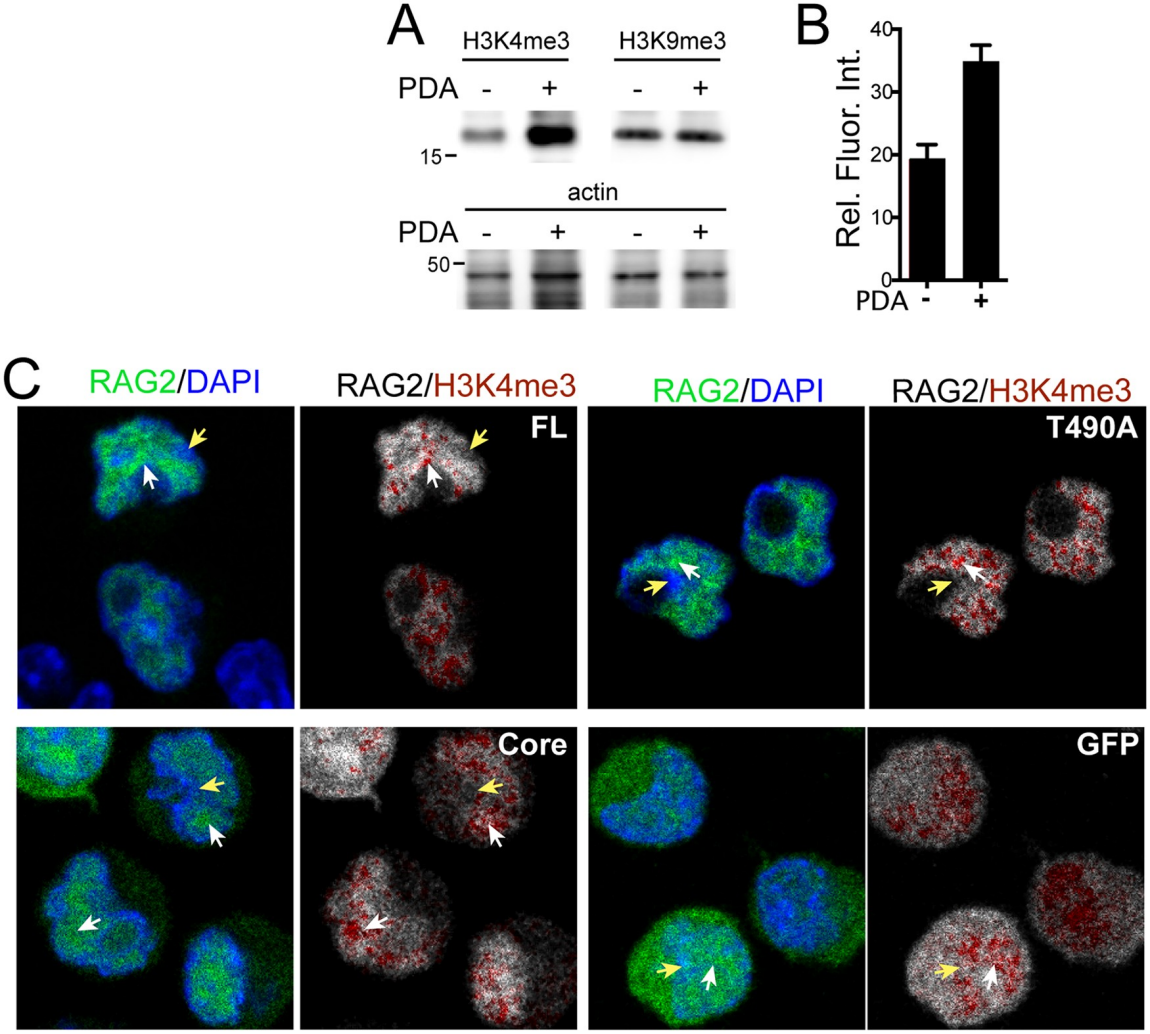
Visualization of GFP-RAG2 proteins in PDA-treated pre-B cells. **(A)** Immunoblot measuring H3K4me3 and H3K9me3 from lysates of pre-B cells that were untreated, or grown for 18 h in 10.0 mM PDA. **(B)** The relative fluorescence intensity (Rel. Fluor. Int.) of labeled H3K4me3 measured in confocal images of untreated and PDA-treated pre-B cells. **(C)** Merge *(left)* and overlay *(right)* images of PDA-treated pre-B cells expressing the indicated GFP-RAG2 or control GFP alone. The merge images were generated using the DAPI *(blue)* and GFP *(green)* channels of the sample; the overlay is H3K4me3 label *(red)* on the GFP channel *(grayscale)*. The white and yellow arrows indicate GFP- and DAPI-enriched domains, respectively. The white bar represents 3 μm.

We also performed correlation analysis in pre-B cells that were treated with the cell-permeable histone demethylase inhibitor PDA, which increased the H3K4me3 content of nuclei (Fig 4A & 4B). However, the PDA did not increase the amount of H3K9me3, and the RAG2 proteins remained localized to DAPI-poor domains in the nucleus that contained H3K4me3 (Fig 4C). Interestingly, treatment with PDA increased T490A and FL correlation with H3K4me3 label, but not Core and GFP alone (Fig 3A). The increase in ρ by PDA specific to the FL and T490A is consistent with the understood role of the RAG2 noncore region in mediating interactions with H3K4me3. Finally, the correlation values of the RAG2 proteins with H3K9me3 were similar in all conditions (Fig 3B), and averaged at a value close to that of the GFP control with either H3K4me3 or H3K9me3 (Fig 3A & 3B).

In contrast to wild-type RAG2, the T490A mutant does not undergo degradation following the G1 stage of the cell cycle [22]. Thus, one concern was that elevated T490A correlation with H3K4me3 was due to labeled RAG2 occurring in S phase nuclei. However, ρ for T490A and H3K4me3 was largely unaffected by cyclin D1 expression (S2A Fig), changing at a rate that would not account for the increased values for ρ in the cells that expressed labeled T490A compared to the FL and Core samples. Thus, blocked degradation of the T490A mutant in S phase nuclei does not account for the elevated correlation of T490A with H3K4me3 relative to other RAG2 proteins. Furthermore, we observed minimal correlation between GFP and H3K4me3 intensities and values for rho (all slopes < 0.003) for cells that expressed either the GFP-FL or -T490A construct (S2B Fig), with the intensities of the labeled FL and T490A occurring over a similar range of values. We therefore conclude that the elevated correlation of T490A label with H3K4me3 relative to that of FL is not an artifact of label intensity in either the GFP or H3K4me3 channels.

### RAG2 regions adjoin H3K4me3-enriched foci

**Fig 5:**
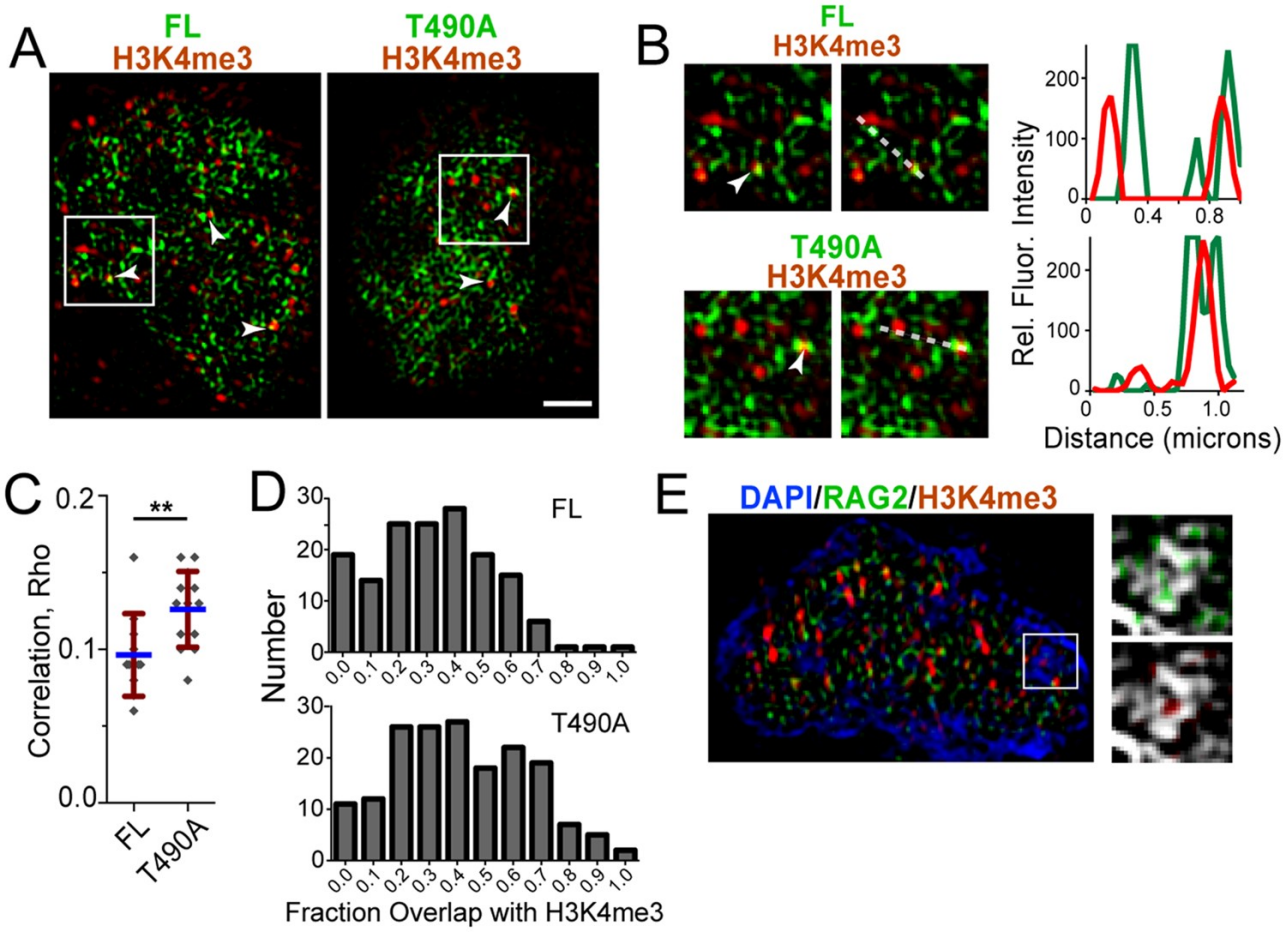
Colocalization of RAG2 with H3K4me3 visualized by superresolution microscopy. **(A)** Merge of SIM images from H3K4me3-stained pre-B cell nuclei that expressed either GFP-FL (*left*) or -T490A (*right*). The arrowheads indicate examples of H3K4me3 puncta that contain adjoining GFP-labeled RAG2. In **(B)** are the regions indicated by boxes in (A). The paired images in each row are identical, with the image on right showing the line used to measure the fluorescence intensities in each channel and plotted in the accompanying graphs *(right)*. **(C)** Correlation values for the GFP-RAG2s and H3K4me3, measured in SIM images of double-labeled pre-B cell nuclei. **(D)** Histograms showing the fraction of pixels in H3K4me3-labeled puncta that contain GFP-FL *(top*) or -T490A *(bottom)*. Individual puncta were outlined in the H3K4me3 channel, and the fraction of pixels that also contain GFP label were scored. **(E)** Merge SIM image of pre-B cell nuclei that expressed GFP-RAG2 *(green)*, and co-stained with DAPI *(blue)* and antibody to H3K4me3 *(red)*. Indicated is a DAPI-enriched region of the nucleus, and adjacent are zoomed regions of the box showing an overlay of GFP *(green)* and H3K4me3 *(red)* labeling on DAPI *(grayscale)*. The white bars represent 1.0 μm.

To better resolve the localization of RAG2 with H3K4me3, we performed **s**tructured **i**llumination **m**icroscopy (SIM) using pre-B cells expressing either GFP-FL or–T490A, and double-labeled with antibody to H3K4me3. With an *x-y* resolution of approximately 100 nm, SIM provides at least a 2-fold improvement in resolution compared to confocal microscopy [29]. In Fig 5A and 5B are merge images of SIM data collected for the labeled RAG2 proteins (green) and H3K4me3 (red). These data show that the H3K4me3 is localized to puncta, and that the labeled RAG2 proteins adjoin, and in some instances overlap, the H3K4me3 puncta (white arrowheads, Fig 5A and 5B). The T490A overlapped with the H3K4me3 puncta more than the FL, as evidenced by a larger correlation coefficient (Fig 5C), and a larger amount of overlap of the GFP signal with H3K4me3 puncta (Fig 5D). Finally, in Fig 5E is an example where RAG2 and H3K4me3 abutted domains of condensed chromatin labeled with DAPI. Localization of H3K4me3 and RAG2 to the periphery of heterochromatin is consistent with the model where active chromatin borders inactive heterochromatin [10]. Other regions containing RAG2 and H3K4me3 that do not contain heterochromatin may represent an interchromatin compartment containing euchromatin together with transcription factories and other DNA/RNA-protein complexes.

### T490A diffuses at a slower rate than FL and Core in the pre-B cell nucleus

**Fig 6:**
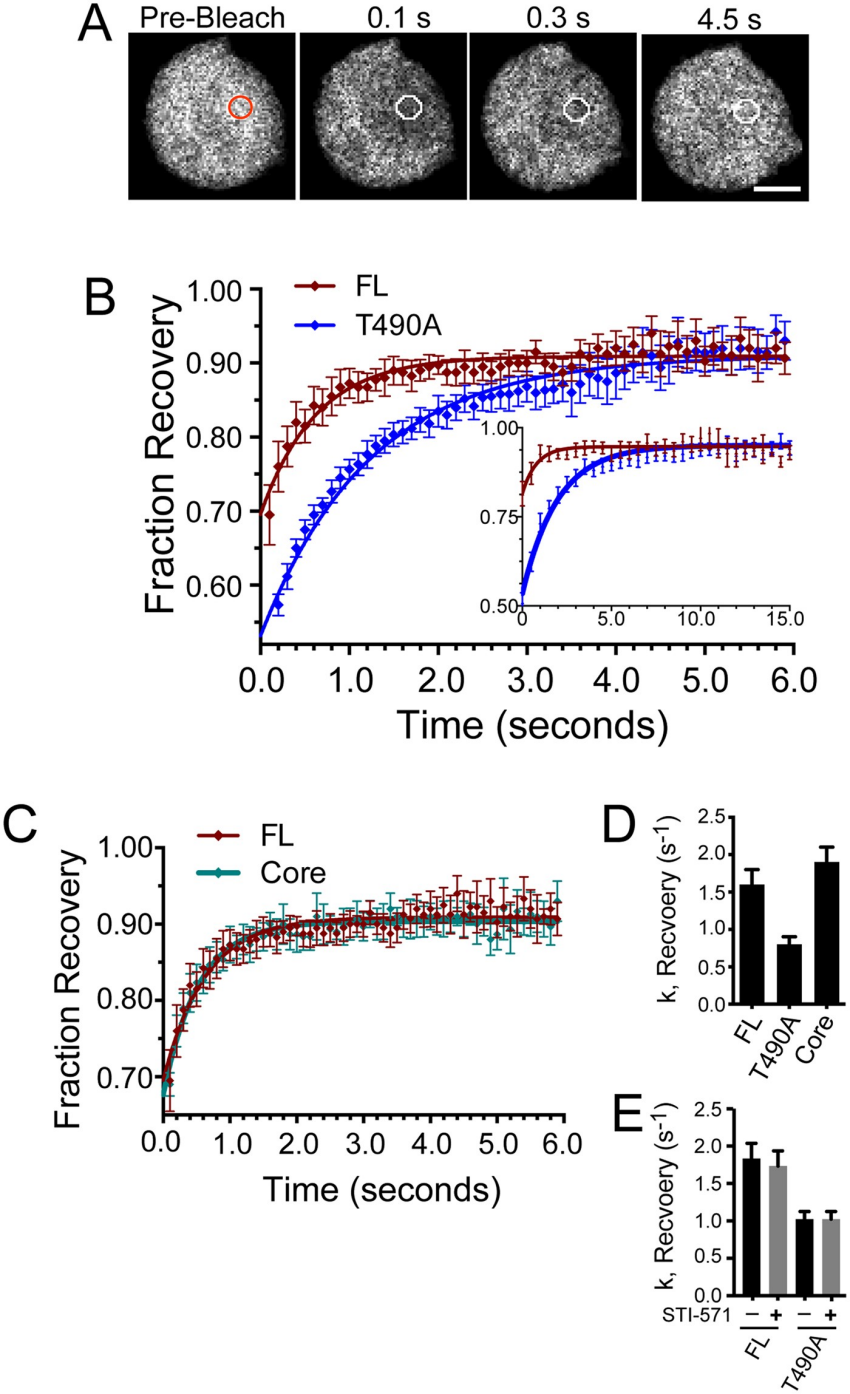
The T490A mutation reduces RAG2 rate of diffusion in the nucleus. **(A)** A representative FRAP measurement of GFP-FL in a pre-B cell. The circles indicate the region of photobleach in the pre-bleach image *(red circle)* and post-bleach images *(white circles)*. The white bar indicates 3.0 μm. **(B)** Recovery curves for GFP-FL *(red)* and -T490A *(blue)* from FRAP of labeled pre-B cell nuclei. Images were collected every 0.1 s following photobleaching. The inset is a recovery curve where the time interval was increased from 0.1 s to 0.5 s, and the measurement extended to 15.0 s. **(C)** Recovery curves for GFP-FL *(red)* and -Core *(black)* determined from FRAP of labeled pre-B cell nuclei. In (B) and (C), the error bars represent SEM, calculated from the averaged data of each time point from five separate trials. Images were collected every 0.1 s following photobleaching. **(D and E)** Averaged rate constants (± SEM) for recovery of fluorescence determined by fitting fluorescence intensity values to a one-component exponential recovery curve. All data are from RAG2^-/-^ pre-B cells that expressed the indicated GFP-RAG2 fusion protein.

To determine if altering the RAG2 noncore domain affected its diffusion within the nucleus, we measured FRAP in pre-B cell nuclei of cells that expressed the GFP-labeled FL, T490A, or Core. In Fig 6A is an example of a FRAP measurement using a cell that expressed GFP-FL. In these experiments, a region of the nucleus was photobleached (circle) by a brief pulse of laser illumination, followed by image acquisition every 0.1 s to follow recovery of fluorescence in the bleached area. Plotting the percent recovery of fluorescence in the photobleached region versus time and fitting these data to an exponential recovery yields the rate of recovery of the fluorescently labeled protein [30], and this is proportional to the rate of diffusion (D) [31]. The amount of the recovery represents the mobile fraction of labeled molecules within the bleached spot.

Plotted in Fig 6B are recovery curves for GFP-FL and-T490A, and represents the averaged results from three or more separate trials, with each trial consisting of eight or more separate FRAP measurements. The inset in Fig 6B are curves from experiments where the fluorescence recovery was extended to 15.0 sec, thus showing the recovery continued to plateau when the experiment was extended to longer time points. The averaged recovery curve for GFP-Core is plotted in Fig 6C together with that for GFP-FL, showing that the recovery curves for these proteins are almost indistinguishable. Table 1 lists the rate and percent recovery determined from the fitted curves for each protein. Summarizing, each of the RAG2 proteins exhibited essentially complete recovery, yet FL and Core had a significantly greater rate of recovery than T490A. For example, the rate constant for T490A was one-half that of FL and Core (Table 1 and Fig 6D). Consistent with this finding, the y-intercept in the recovery curves for the GFP-T490A was less than either the FL or Core proteins. FL and T490A are bleached to a similar amount (S3 Fig), yet the reduced rate of recovery for the T490A protein allowed capture of recovery in initial frames that was less complete than that of the GFP-FL and Core proteins. Finally, induction of RAG1 expression by treating cells with STI-571 had no effect on either the rate or the amount of recovery for both the FL and T490A constructs (Fig 6E and S4 Fig). Thus, interactions of RAG2 proteins with nuclear proteins reported by FRAP measurements are not affected by STI-571-induced expression of RAG1.

**Table 1:** Rate constant and percent recovery of GFP-labeled RAG2 proteins determined by fitting recovery curves to an exponential function.

	FL	T490A	Core	W453A, T490A
k (s^-1^)	1.6 ± 0.2	0.8 ± 0.1	1.9 ± 0.2	1.8 ± 0.2
% Recovery	91 ± 0.0	91 ± 0.0	90 ± 0.0	91 ± 1.0

### The differing localization and mobility properties between T490A and FL are dependent on the PHD

**Fig 7:**
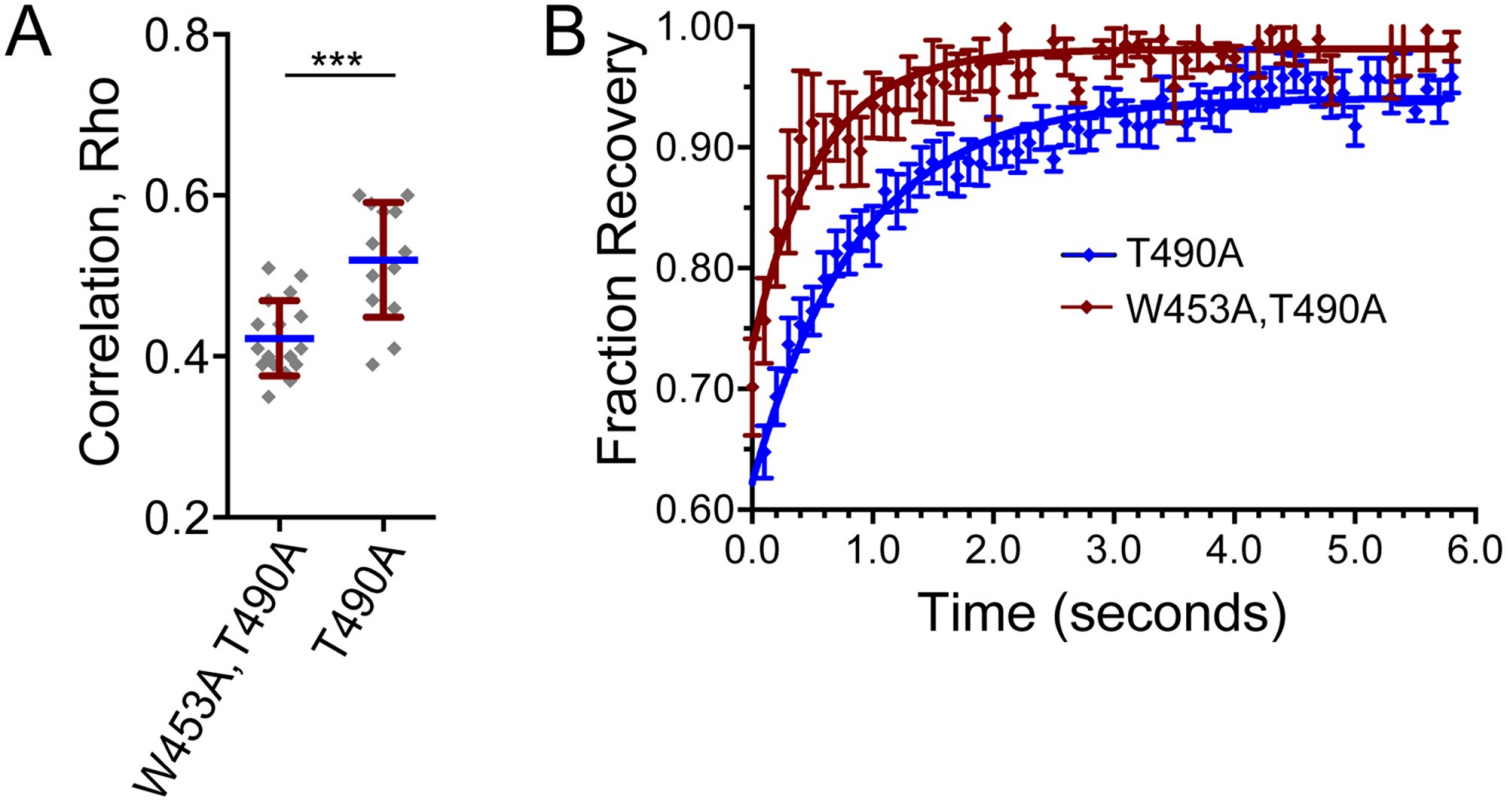
W453A blocks T490A-dependent changes in RAG2 colocalization with H3K4me3 and intranuclear mobility. **(A)** Colocalization of W453A,T490A and T490A with H3K4me3 quantified in populations of double-labeled cells. The correlation coefficient was measured as described for Fig 3, with each symbol representing ρ determined for an individual cell **(B)** FRAP analysis of GFP-labeled W453,T490A and T490A. The error bars represent SD and SEM in (A) and (B), respectively.

To test if the PHD affected the unique localization and mobility properties of T490A, we generated the double mutant W453A,T490A, which eliminates binding of RAG2 to H3K4me3 [18]. Strikingly, the double mutant exhibited significantly less colocalization with H3K4me3 than T490A (Fig 7A), and a rate of fluorescence recovery most similar to that of the FL and Core proteins (Table 1 and Fig 7B). The slower rate of T490A diffusion compared to FL and W453A,T490A is consistent with the T490A mutation altering RAG2 interactions with binding partners in the nucleus, such as H3K4me3, through a PHD-dependent interaction.

## Discussion

The V(D)J recombinase is composed of the proteins RAG1 and RAG2, which together function in the incision of specific genetic loci for assembly of AR genes [1, 2]. Enzymatic activity of the recombinase complex is restricted to RAG1, yet RAG2 is essential for relieving an autoinhibition of the recombinase through binding to H3K4me3 by a PHD region in the RAG2 noncore domain [11–13]. Since H3K4me3 populates sites of recombination in the AR locus, RAG2 noncore interactions with H3K4me3 will both target the recombinase to potential recombination sites, and activate the complex for V(D)J recombination. Previous studies show RAG2 is sequestered from transcriptionally inactive heterochromatin within the nucleus [8, 9], but the effect of altering the noncore domain on RAG2 localization and dynamics is not understood. Here, we used analytical imaging approaches to measure the role of the noncore domain in RAG2 localization and dynamics to elucidate its role in targeting RAG2 to chromatin in pre-B cell nuclei.

In pre-B cells, we observed that RAG2 occupied DAPI-poor regions in the nucleus, concentrating in channels between heterochromatin-rich domains. Within the RAG2-labeled regions was H3K4me3, while most of the H3K9me3 marker for inactive chromatin occurred in the DAPI-enriched zones that included the nuclear periphery (Figs 1 and 2). Measuring mutants of the noncore region, we observed that T490A exhibited significantly greater colocalization with H3K4me3 than FL (Fig 3), and this corresponded to a lower rate of diffusion compared to the other RAG2 proteins (Fig 6). In contrast, FL RAG2 showed dynamics and localization with H3K4me3 similar to Core RAG2, which does not have a PHD region for interacting with H3K4me3. However, elevating nuclear H3K4me3 levels by treating cells with PDA increased both FL and T490A colocalization with H3K4me3, but not Core.

**Fig 8:**
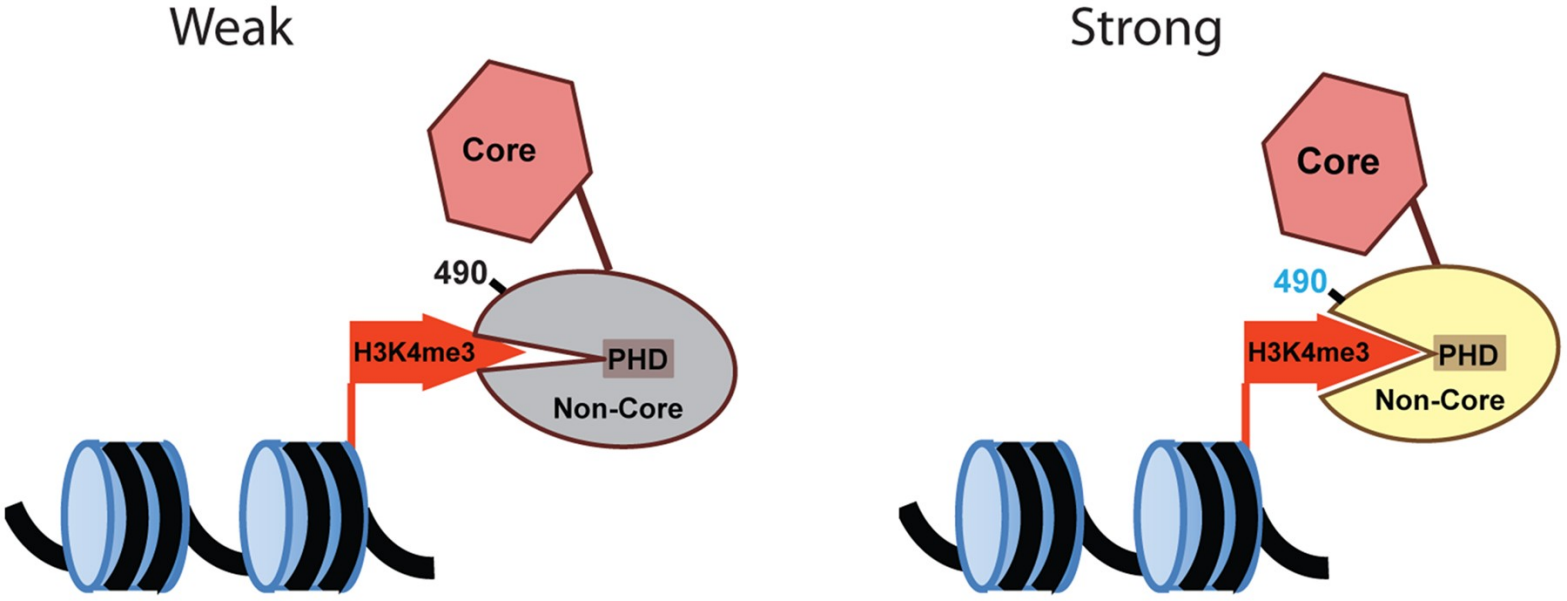
Model for Thr^490^-dependent regulation of RAG2 interactions with H3K4me3. RAG2 alternates between weak and strong interactions with H3K4me3 via a PHD in its noncore region (indicated), and mediated by allosteric changes in the noncore region localized at residue 490. In wild-type protein, allosteric changes may occur through post-translational modifications of Thr490, which are mimicked by the T490A mutation.

Our findings suggest that in steady-state conditions, the noncore region of RAG2 is in a state that disfavors stable interactions with H3K4me3 to result in colocalization and dynamics most similar to Core, and that is reversed in conditions that elevate the H3K4me3 content of cell nuclei. Conversely, the elevated colocalization of the T490A mutant with H3K4me3 and its lower rate of mobility relative to FL suggest this mutant adopts a separate structure or conformation that enhances interactions with H3K4me3 and perhaps other nuclear proteins. Accordingly, we propose a model where Thr^490^ functions in allosteric control of RAG2 interactions with H3K4me3 by modulating the conformation of the noncore region (Fig 8). Importantly, Thr^490^ is proximal to the PHD in the noncore, which mediates RAG2 interactions with H3K4me3. The allosteric control would likely involve the PHD region, evidenced by our data showing that mutating Trp^453^ in T490A resulted in a double mutant with properties most similar to that of FL rather than T490A. Furthermore, unidentified triggers may cause a Thr^490^-dependent change in the RAG2 noncore to a conformation that disfavors interaction with H3K4me3. The trigger for the conformation change is unclear, but may include phosphorylation or other post-translational modification, which will be blocked by the T490A mutation. Interestingly, it was previously shown that methylation of K507 in human RAG2 alters it intranuclear distribution pattern in transiently transfected nonlymphoid cells [26], further supporting that post-translational modification of the noncore region of RAG2 can have profound consequences on its fundamental properties. Further studies are necessary, however, to resolve separate conformations of RAG2 noncore, the triggers that induce switching between possible conformations, and the effect of these changes on RAG2 localization and dynamics.

Studies of nuclear structure suggest heterochromatin coated with transcriptionally active euchromatin occur together as islands within interchromatin channels (IC) occupied by freely diffusing molecules [10]. Nuclear proteins that interact with active chromatin, such as transcription factors and the V(D)J recombinase, are thought to diffuse through the interchromatin channels until contacting binding partners within open chromatin regions that line heterochromatin. Consistent with this model, we observed RAG2 colocalization with H3K4me3 included regions that abutted DAPI-labeled heterochromatin (Fig 5E). The positioning of active AR loci that undergo recombination relative to this complex network of DAPI-rich versus RAG2-labeled regions remains to be determined.

Interestingly, superresolution imaging showed that RAG2 occurred adjacent to and overlapping with H3K4me3-containing puncta. The greater overlap of T490A versus FL for the puncta is consistent with T490A assuming a conformation that favors more stable interactions with chromatin. The H3K4me3 puncta may include multiple H3K4me3-modified nucleosomes and other factors that constitute transcription factories important for regulating gene expression [32], and further study is necessary to resolve their composition.

Treating cells with STI-571 to increase RAG1 expression had no effect on FL and T490A RAG2 mobility (Fig 6E and S4 Fig). Based on these results, it is unlikely that RAG1 affects RAG2 interactions with H3K4me3 since targeting of RAG2 to H3K4me3-modified chromatin is independent of RAG1 [15]. Previous ChIP-seq studies indicated that RAG1 was present with RAG2 at H3K4me3-enriched sites [16], and thus we predict RAG1 is proximal with RAG2 to H3K4me3 puncta throughout the nucleus.

Our FRAP data show that essentially all of the RAG2 is mobile, and the half-time for the recoveries indicate that interactions with large binding partners, such as H3K4me3-modified nucleosomes, are not longer than several seconds in duration. These dynamics are consistent with the entire RAG2 pool rapidly and simultaneously sampling H3K4me3 sites throughout the genome, such as indicated by ChIP-seq data [15, 16]. Notably, viable RSS sites are preferentially depleted at genomic regions containing H3K4me3 outside of AR loci [16, 33]. Thus, while RAG2 performs a whole genome scan of open chromatin sites through interactions with H3K4me3, it is primarily the H3K4me3 sites in AR loci proximal to RSSs that allow for recombinase activity. Moreover, H3K4me3-containing puncta in regions proximal to the AR may also contain factors that favor recombinase activity at these sites. However, whether the H3K4me3-labeled puncta proximal to AR are unique in a manner that supports V(D)J recombination remains to be determined.

The two-fold difference in recovery rate for FL versus T490A may reflect longer-lived interactions between T490A and immobile binding partners, such as modified histones. Supporting this interpretation is that the slower recovery rate for T490A is dependent on a functional PHD. Alternatively, the slower rate of recovery may reflect association of the T490A mutant with mobile complexes that are significantly larger in size than RAG2 alone. In this case, the difference in recovery rates would reflect T490A association with protein complexes that are approximately 8-fold larger in size than those containing FL, assuming the complexes are spherical in shape [30]. These two interpretations regarding the difference in recovery rates between FL and T490A are not mutually exclusive, and further study is necessary to distinguish which better reflects the properties of RAG2 in these conditions.

In summary, we show here evidence that RAG2 is distributed throughout the pre-B cell nucleus in DAPI-poor regions that contain H3K4me3 and channel between heterochromatin-rich domains containing H3K9me3. Our data also shows evidence where Thr^490^ modulates RAG2 interactions with H3K4me3, and that these interactions occur at the interface of discrete nanoscopic complexes containing RAG2 and H3K4me3. Based on our findings, we propose a model where the RAG2 noncore domain undergoes a conformational switch between states that favor and oppose interactions with H3K4me3, and that Thr^490^ regulates this switching. Mechanisms that modulate Thr^490^-mediated changes in the RAG2 noncore structure are predicted to be important towards triggering, or conversely suppressing, RAG recombinase activity during lymphocyte development.

## Supporting information

S1 Fig GFP-RAG2 expression and activity in V(D)J recombination. **(A)** Immunoblot of A70 cell lysates and lysate of a pre-B cell clone expressing GFP-FL. Following transfer, the membrane was probed using monoclonal antibody to RAG2. 5.0 μM STI-571 was added to A70 cells to induce expression of RAG2. **(B)** Extrachromosomal plasmid recombination assay. HEK293T cells were transiently transfected with the recombination substrate pSF299 [34], along with plasmids encoding for MBP-core RAG1 [35] and either GFP alone or fused to RAG2 (Core, FL, or T490A). Plasmid DNA was isolated 72 hrs post-transfection according to the Hirt procedure [36], and total pSF299 plasmid substrate and recombined signal joints amplified by PCR and relative recombination efficiencies determined. Signal joint amplicon identity was confirmed with an ApaLI restriction digest (not shown). **(Top)** Representative gel image of semi-quantitative PCR of signal joint amplicons separated on an 8% polyacrylamide gel and stained with SYBR gold. Signal joints were amplified from serial 2-fold dilutions of recombined pSF299 plasmids isolated from cells expressing MBP-core RAG1 and GFP (lanes 2–4), core (lanes 4–6), FL (lanes 7–9), or T490A (lanes 10–12). The signal joint amplicon is 167 base pairs (indicated by arrow). **(Bottom)** PCR amplicon fluorescence intensities were quantified and averaged for each of three replicates. The mean intensity for FL-RAG2 was normalized to 1.0. Error bars depict SD, n = 3.
(TIF)

S2 Fig Analysis of the effects of cell cycle and protein expression on GFP-T490A colocalization with H3K4me3. **(A)** Plot of correlation values for GFP-T490A and H3K4me3 vs. Cyclin D1 intensity measured in confocal images of pre-B cells. Shown are the results from a linear regression analysis with the 95% confidence interval for the curve fit. The slope of the line from the linear regression analysis equals 0.0001. **(B)** Correlation values plotted versus the mean fluorescence intensity of the cell in the GFP *(top)* and H3K4me3 *(bottom)* channels of pre-B cells that expressed either GFP-T490A or GFP-FL. The slopes ranged between 0.0004 and 0.0007 for the plots of GFP intensity (*top*), and equaled 0.003 for both plots of H3K4me3 intensity (*bottom*).
(TIF)

S3 Fig Separate GFP-RAG2s undergo a similar amount of bleaching during FRAP measurements. **(A)** The pre-bleach and first frame following photobleaching of FL *(top)* and T490A *(bottom)* in *RAG2*^*-/-*^ pre-B cells. To block recovery following photobleaching, the cells were fixed with paraformaldehyde prior to measurement. In **(B)** is plotted the fraction of signal in the bleach spot in the first frame following photobleaching relative to the signal in the spot in the prebleach image, measured in a set of FL and T490A-expressing cells (n = 6). The white bar in (A) represents 3 μm.
(TIF)

S4 Fig Induction of RAG1 expression by STI-571 does not affect RAG2 FL and T490A mobility in the pre-B cell nucleus. **(A)** Induction of endogenous RAG1 expression levels with STI-571 treatment as detected by immunoblotting with rabbit monoclonal antibody to RAG1 (clone EPRAGR1, Abcam, Cambridge, MA). Lane 1 is a negative control, consisting of whole cell extract from a v-abl *RAG1*^*-/-*^ pro-B cell line that was generously provided by Luigi Notarangelo. Lanes 2 and 3 show RAG1 detected from whole cell extracts of GFP-FL RAG2 expressing cells that were either untreated, or treated overnight with 5 μM STI-571 as indicated. The GAPDH loading control is shown beneath each lane. **(B)** FRAP recovery curves of FL (*top*) and T490A (*bottom*) in cells that were either untreated, or treated with 5 μM STI-571 overnight prior to measurement. The curves represent fits from 7 or more measurements; the error bars are SEM. The recovery rates were 1.8 ± 0.2 s^-1^ and 1.7 ± 0.2 s^-1^ for FL control and with STI-571, respectively. For T490A, the recovery rate was 1.0 ± 0.1 s^-1^ for both samples.
(TIF)
