# Supplementary figures and images for: RAG2 localization and dynamics in the pre-B cell nucleus
Source: PLoS One. 2019 May 10;14(5):e0216137. doi: 10.1371/journal.pone.0216137 (PMC6510410; doi:10.1371/journal.pone.0216137)

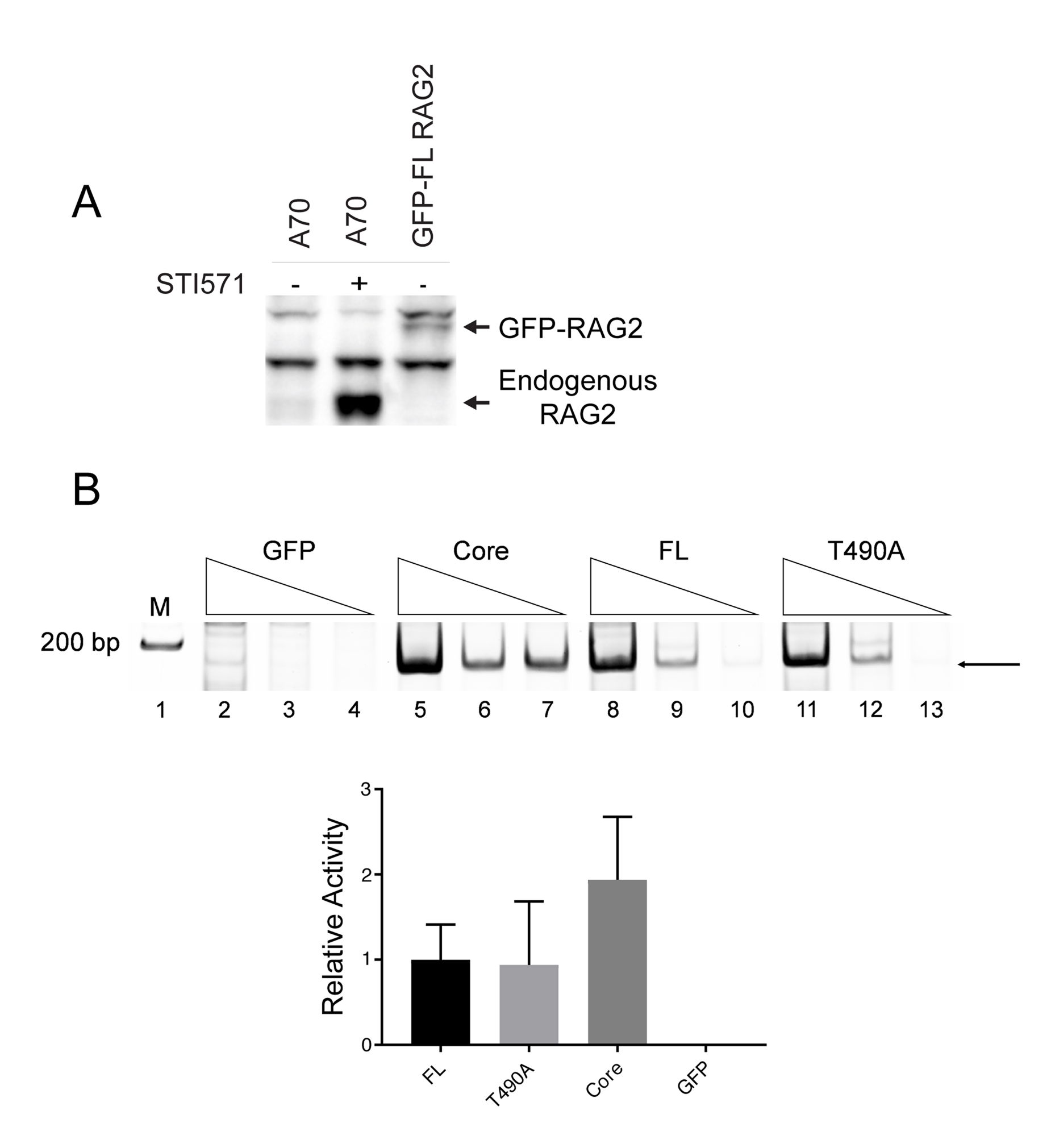

Supplement: S1 Fig — (A) Immunoblot of A70 cell lysates and lysate of a pre-B cell clone expressing GFP-FL. Following transfer, the membrane was probed using monoclonal antibody to RAG2. 5.0 μM STI-571 was added to A70 cells to induce expression of RAG2. (B) Extrachromosomal plasmid recombination assay. HEK293T cells were transiently transfected with the recombination substrate pSF299 [34], along with plasmids encoding for MBP-core RAG1 [35] and either GFP alone or fused to RAG2 (Core, FL, or T490A). Plasmid DNA was isolated 72 hrs post-transfection according to the Hirt procedure [36], and total pSF299 plasmid substrate and recombined signal joints amplified by PCR and relative recombination efficiencies determined. Signal joint amplicon identity was confirmed with an ApaLI restriction digest (not shown). (Top) Representative gel image of semi-quantitative PCR of signal joint amplicons separated on an 8% polyacrylamide gel and stained with SYBR gold. Signal joints were amplified from serial 2-fold dilutions of recombined pSF299 plasmids isolated from cells expressing MBP-core RAG1 and GFP (lanes 2–4), core (lanes 4–6), FL (lanes 7–9), or T490A (lanes 10–12). The signal joint amplicon is 167 base pairs (indicated by arrow). (Bottom) PCR amplicon fluorescence intensities were quantified and averaged for each of three replicates. The mean intensity for FL-RAG2 was normalized to 1.0. Error bars depict SD, n = 3. (TIF) [file pone.0216137.s001.tif]

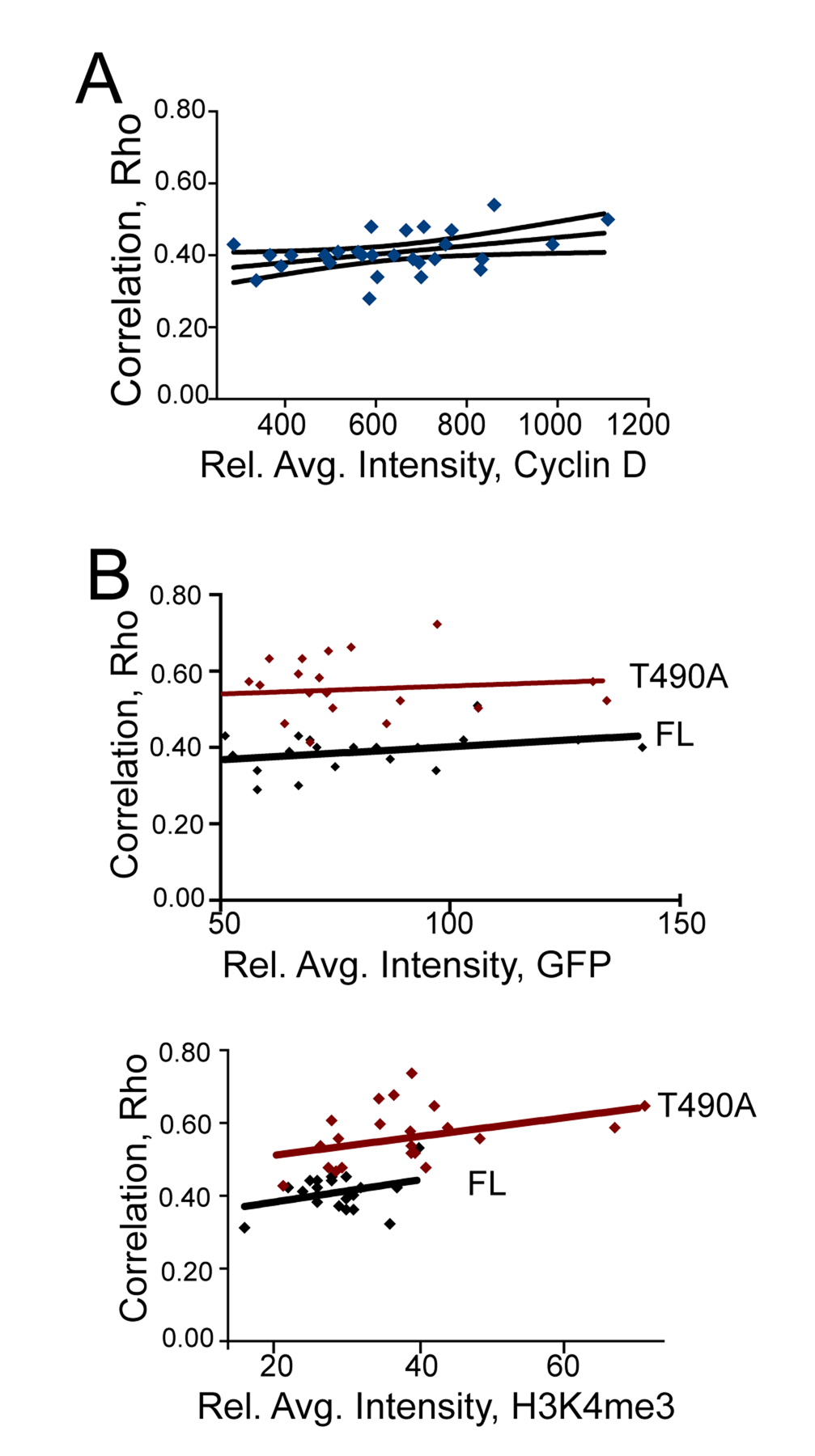

Supplement: S2 Fig — (A) Plot of correlation values for GFP-T490A and H3K4me3 vs. Cyclin D1 intensity measured in confocal images of pre-B cells. Shown are the results from a linear regression analysis with the 95% confidence interval for the curve fit. The slope of the line from the linear regression analysis equals 0.0001. (B) Correlation values plotted versus the mean fluorescence intensity of the cell in the GFP (top) and H3K4me3 (bottom) channels of pre-B cells that expressed either GFP-T490A or GFP-FL. The slopes ranged between 0.0004 and 0.0007 for the plots of GFP intensity (top), and equaled 0.003 for both plots of H3K4me3 intensity (bottom). (TIF) [file pone.0216137.s002.tif]

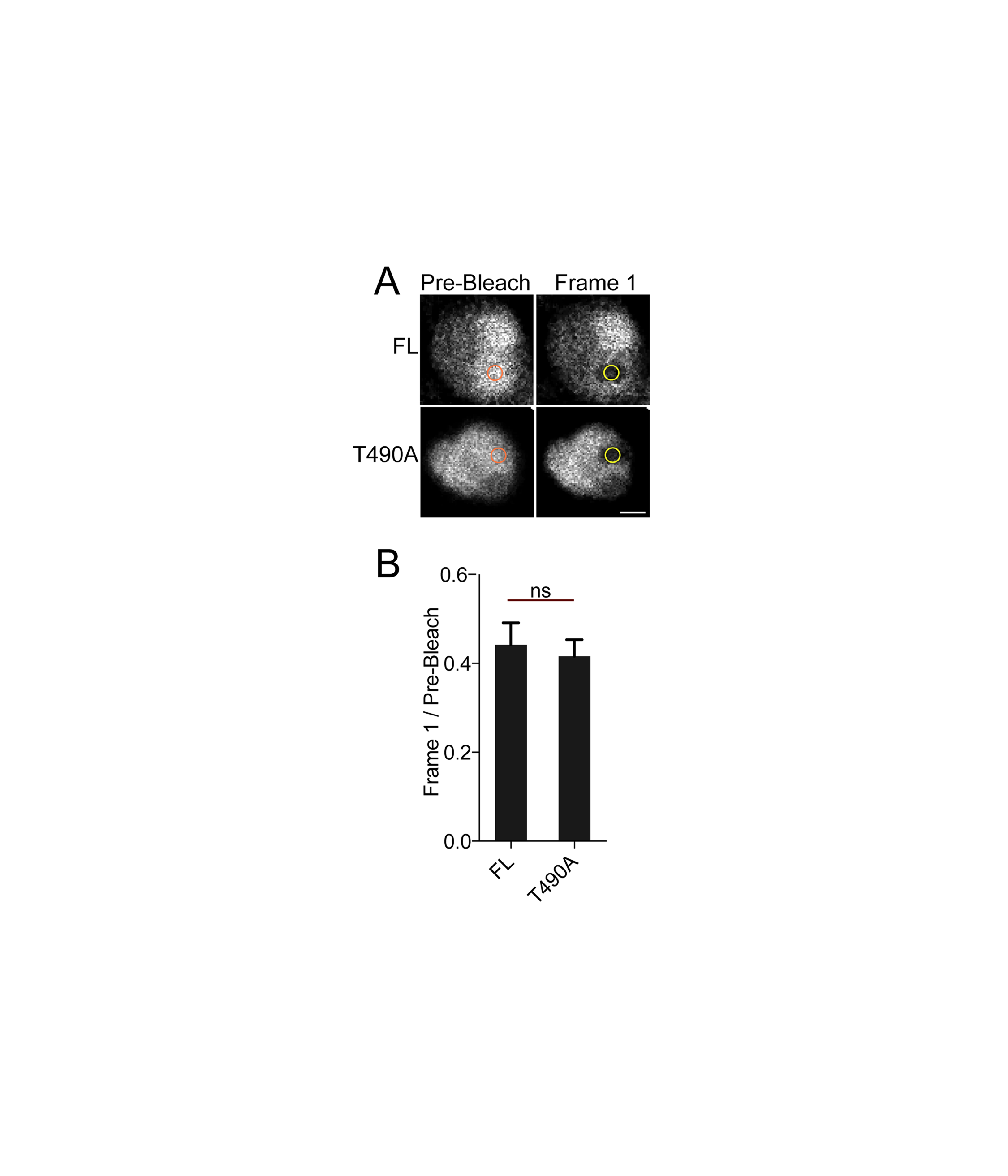

Supplement: S3 Fig — (A) The pre-bleach and first frame following photobleaching of FL (top) and T490A (bottom) in RAG2-/- pre-B cells. To block recovery following photobleaching, the cells were fixed with paraformaldehyde prior to measurement. In (B) is plotted the fraction of signal in the bleach spot in the first frame following photobleaching relative to the signal in the spot in the prebleach image, measured in a set of FL and T490A-expressing cells (n = 6). The white bar in (A) represents 3 μm. (TIF) [file pone.0216137.s003.tif]

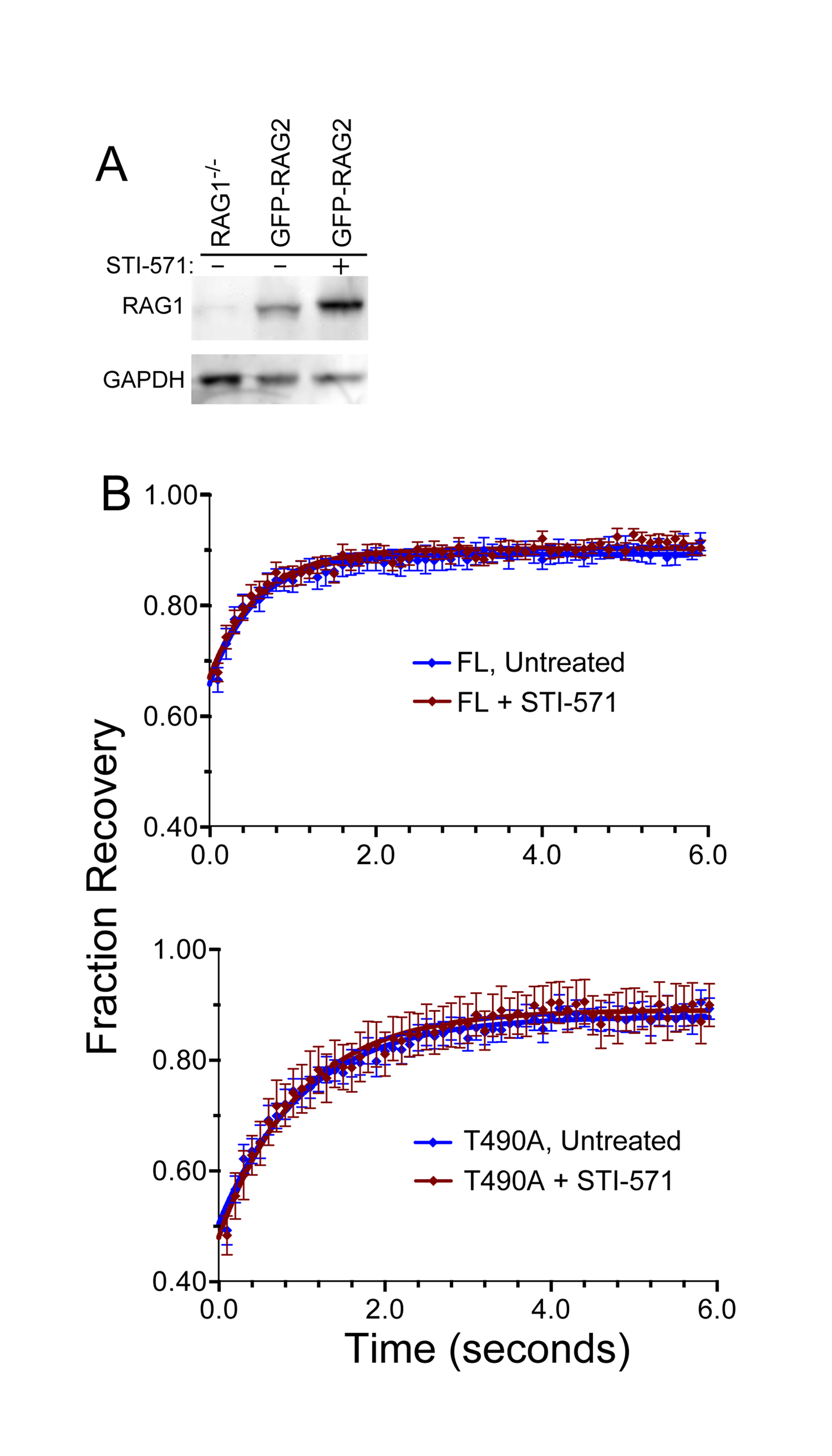

Supplement: S4 Fig — (A) Induction of endogenous RAG1 expression levels with STI-571 treatment as detected by immunoblotting with rabbit monoclonal antibody to RAG1 (clone EPRAGR1, Abcam, Cambridge, MA). Lane 1 is a negative control, consisting of whole cell extract from a v-abl RAG1-/- pro-B cell line that was generously provided by Luigi Notarangelo. Lanes 2 and 3 show RAG1 detected from whole cell extracts of GFP-FL RAG2 expressing cells that were either untreated, or treated overnight with 5 μM STI-571 as indicated. The GAPDH loading control is shown beneath each lane. (B) FRAP recovery curves of FL (top) and T490A (bottom) in cells that were either untreated, or treated with 5 μM STI-571 overnight prior to measurement. The curves represent fits from 7 or more measurements; the error bars are SEM. The recovery rates were 1.8 ± 0.2 s-1 and 1.7 ± 0.2 s-1 for FL control and with STI-571, respectively. For T490A, the recovery rate was 1.0 ± 0.1 s-1 for both samples. (TIF) [file pone.0216137.s004.tif]
